# A new technique for performing interstitial implants for gynecologic malignancies using transvaginal ultrasound guidance

**DOI:** 10.3389/fonc.2022.858620

**Published:** 2022-08-11

**Authors:** Xiao-Jing Yan, Yi Yang, Xi Chen, Shi-Guang Wang, Shu-Huai Niu, Hui-Xian Niu, Hong Liu

**Affiliations:** ^1^ Gynecology and Obstetrics Ultrasound Department, The Fourth Hospital of Hebei Medical University, Shijiazhuang, China; ^2^ Department of Gynecologic Oncology, The Fourth Hospital of Hebei Medical University, Shijiazhuang, China

**Keywords:** interstitial brachytherapy radiotherapy, ultrasound guidance device, gynecological oncology, new technique, performing interstitial implants

## Abstract

**Objectives:**

This study concerns a new technique that aims to achieve precise interstitial brachytherapy of pelvic recurrent tumors under transvaginal ultrasound (US) guidance, enhance the conformity index of the brachytherapy (BT), and improve the curative effect of radiotherapy for gynecological oncology patients with pelvic relapse.

**Methods:**

A real-time transvaginal US-guided interstitial implant device was developed to assist in implant BT. Prior to implant brachytherapy, the size and location of the tumor in the pelvis and the interrelationship with adjacent organs were first assessed with intracavitary ultrasound. The transvaginal US-guided interstitial implant device was then placed on the endoluminal ultrasound probe, the probe was oriented intravaginally to determine a safe needle path, the implant needle was placed into the needle passage of the device, and the implant needle was inserted into the tumor tissue in the direction guided by the ultrasound puncture guide line. After the implant needle was placed in place, the cover of the transvaginal US-guided interstitial implant device was opened perpendicular to the ultrasound probe, and the needle was separated from the ultrasound probe smoothly, and then the cover was re-covered for subsequent implantation.

**Results:**

In this study, 56 patients who underwent real-time transvaginal ultrasound-guided implantation for gynecologic oncology were enrolled, and insertion of 736 implant needles was completed. Among them, 13 patients had recurrent pelvic tumors after cervical cancer surgery and 6 patients had recurrent pelvic tumors after endometrial cancer surgery. Thirty-two patients who underwent radical radiation therapy for cervical cancer did not have adequate regression of parametrial invaded tissue after completion of standard EBRT treatment; and 5 patients had recurrent tumors in the radiation field after previous standard course of pelvic radiotherapy. The accuracy of the implant therapy was improved. The radiotherapy dose for recurrent pelvic masses was successfully increased, and the cumulative dose of external irradiation combined with BT was augmented to 80–100 Gy. The use of a new device for transvaginal implant for recurrent masses located in the lateral wall of the pelvic cavity was successful.

**Conclusion:**

This intravascular US-guided interstitial implant device can realize interstitial implant with the shortest path under transvaginal US guidance. With convenient operation, high precision, and good security, the device not only improves the accuracy of implant therapy, but it also reduces the risks of anesthesia and organ injury, so it is suitable for widespread promotion and use.

## Introduction

The treatment of recurrent pelvic tumors is a great challenge for clinicians regardless of whether surgery or radiotherapy is performed. The latter plays a very important role in gynecological oncology treatment (1–3), and its advantages over surgical treatment are that it reduces trauma, avoids the risks of anesthesia, and preserves the integrity of adjacent organs. With the widespread development of medical image-guided three-dimensional (3D) interstitial implant radiotherapy, the therapeutic effect in patients for whom intensity-modulated radiation therapy is unsuitable has been greatly improved. The precision of interstitial brachytherapy (ISBT) has allowed for an increase in the radiotherapy dose of pelvic masses, thus improving the radiotherapy efficacy for these patients (4). However, radiotherapy doctors have always had to address the difficulty of choosing the best implanting needle placement path to achieve precise positioning and needle placement for small pelvic lesions and masses near the pelvic wall and realize better target conformal implant therapy. Published studies to date focus on 3D-printing non-coplanar template (PNCT)-assisted computed tomography (CT)-guided ISBT-technology or bare-handed operator implantation (5–7). 3D-PNCT technology is not only time-consuming, but it also requires anesthesia, increasing the patient’s pain and the level of risk. On the other hand, bare-handed implantation is difficult, requiring highly-experienced clinicians, and, in particular, the level of precision is low, and repeatability is poor, for small pelvic lesions. In addition, neither the 3D-PNCT positioning technique nor bare-handed implantation can be done under visual conditions, so there is a high risk of organ injury (8). Ultrasound (US), however, has some unique advantages in brachytherapy (BT) the most important advantage of ultrasound guidance is its real-time nature., and, thus, it is an important imaging modality in BT. High-dose prostate implant radiotherapy under transrectal US guidance has been widely used, but the same approach using US can also be applied in gynecological tumor therapy (9).

Transvaginal pelvic puncture has the advantages of a shorter path and less pain, and it requires no anesthesia. Traditional transvaginal treatment requires exposure of the vagina with a speculum, but the use of a speculum restricts the operation angle. Even if the needle is tilted to the maximum angle, it cannot approach the area of the pelvic wall. It has therefore generally been believed that transvaginal implant treatment of tumors near the lateral wall of the pelvic cavity is not feasible. Thus, for masses located in the pelvic wall region, performing a transvaginal interstitial implant to achieve a radiotherapy boost has not been considered possible. However, a transvaginal pelvic US examination does not require the use of a speculum, and the elasticity of the vaginal wall can be utilized so that tumors located in various positions within the pelvic cavity can be observed. It was this principle that was used by the gynecologic oncology radiotherapy team at the Fourth Hospital of Hebei Medical University to develop a transvaginal US-guided puncture bracket for puncture and needle placement under real-time US guidance in the treatment of female pelvic tumors with ISBT. The new device successfully solved the above-mentioned clinical difficulties. This paper describes the structure and function of the puncture guide bracket, as well as outlining its preliminary application results in the clinical treatment of gynecological tumors with ISBT.

## Materials and methods

In this study, 56 patients who underwent real-time transvaginal ultrasound-guided implantation for gynecologic oncology in the Department of Gynecologic Oncology at the Fourth Hospital of Hebei Medical University were enrolled, and the insertion of 736 implant needles was completed. Among them, 13 patients had recurrent pelvic tumors after cervical cancer surgery and 6 patients had recurrent pelvic tumors after endometrial cancer surgery. Thirty-two patients who underwent radical radiation therapy for cervical cancer did not have adequate regression of parametrial invaded tissue after completion of standard EBRT treatment(45–50 Gy in 25–28 fractions); and 5 patients had recurrent tumors in the radiation field after previous standard course of pelvic radiotherapy.

The transvaginal US-guided puncture bracket for interstitial implant was made of medical materials, using injection molding, and was produced in strict accordance with the principle and structure characteristics of the patent (Patent No.: ZL 202010532524.0).

### The structure of the guide device

**Figure 1:**
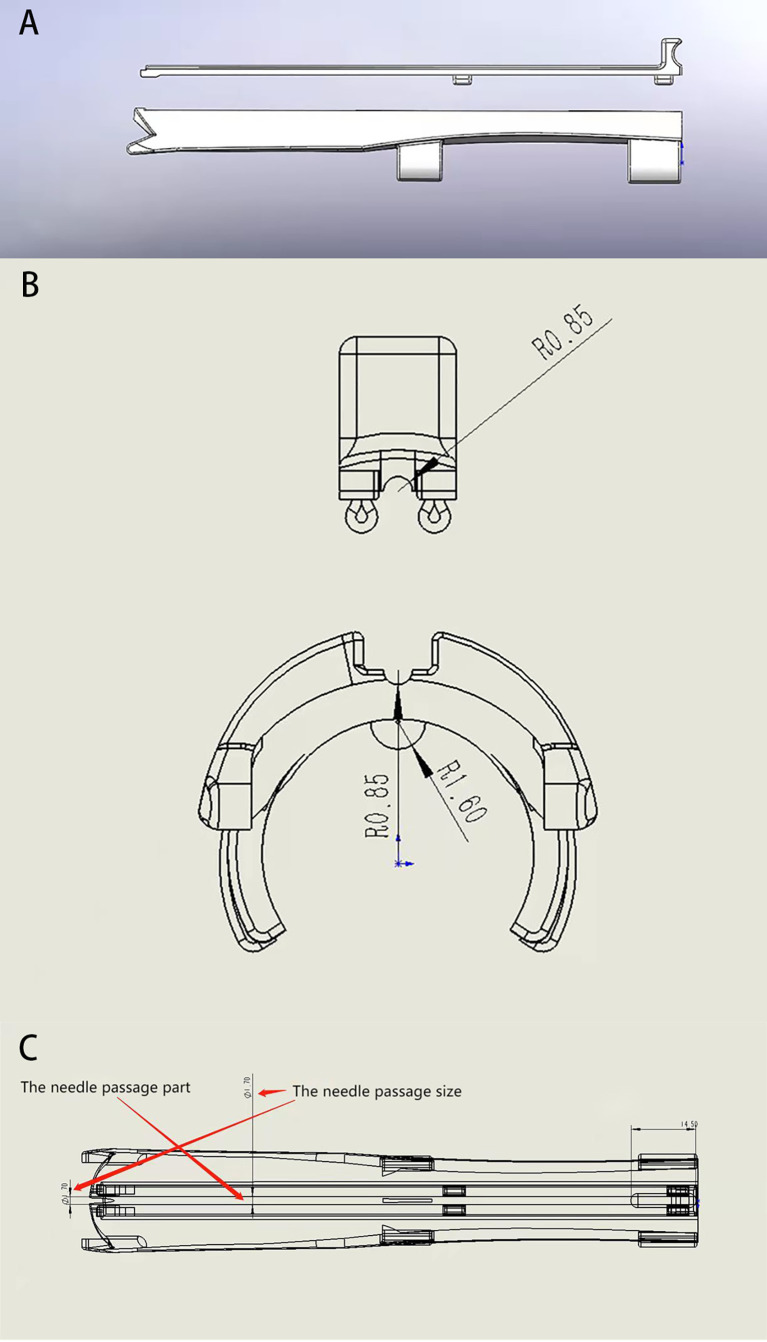
Structure diagram of a transvaginal ultrasound-guided puncture bracket. **(A)** the side of the main body and cover plate of the puncture holder; **(B)** cross section of piercing rack; **(C)** Internal structure of the puncture holder body.

The transvaginal US-guided puncture bracket for interstitial implant consists of a fixed part and a detachable part, the former being the base of the puncture bracket, and the latter the cover plate of the puncture needle (**Figure 1**). The base of the puncture bracket is connected to the transvaginal US probe by mechanical grip force and a convex–concave groove, which is tightly coupled with the ultrasonic probe to fix the needle passage. The base is equipped with a needle passage for the implantation needle, which has a diameter of 1.7 mm, and there are two slide rails on both sides of the passage. The bracket base has four grooves in which to fix the upper detachable cover plate. As a detachable unit, the cover plate can be installed and separated using the slide rail, and the end is designed with a handheld operation apophysis to facilitate separation of the cover plate (**Figure 1**).

### The device capabilities

**Figure 2:**
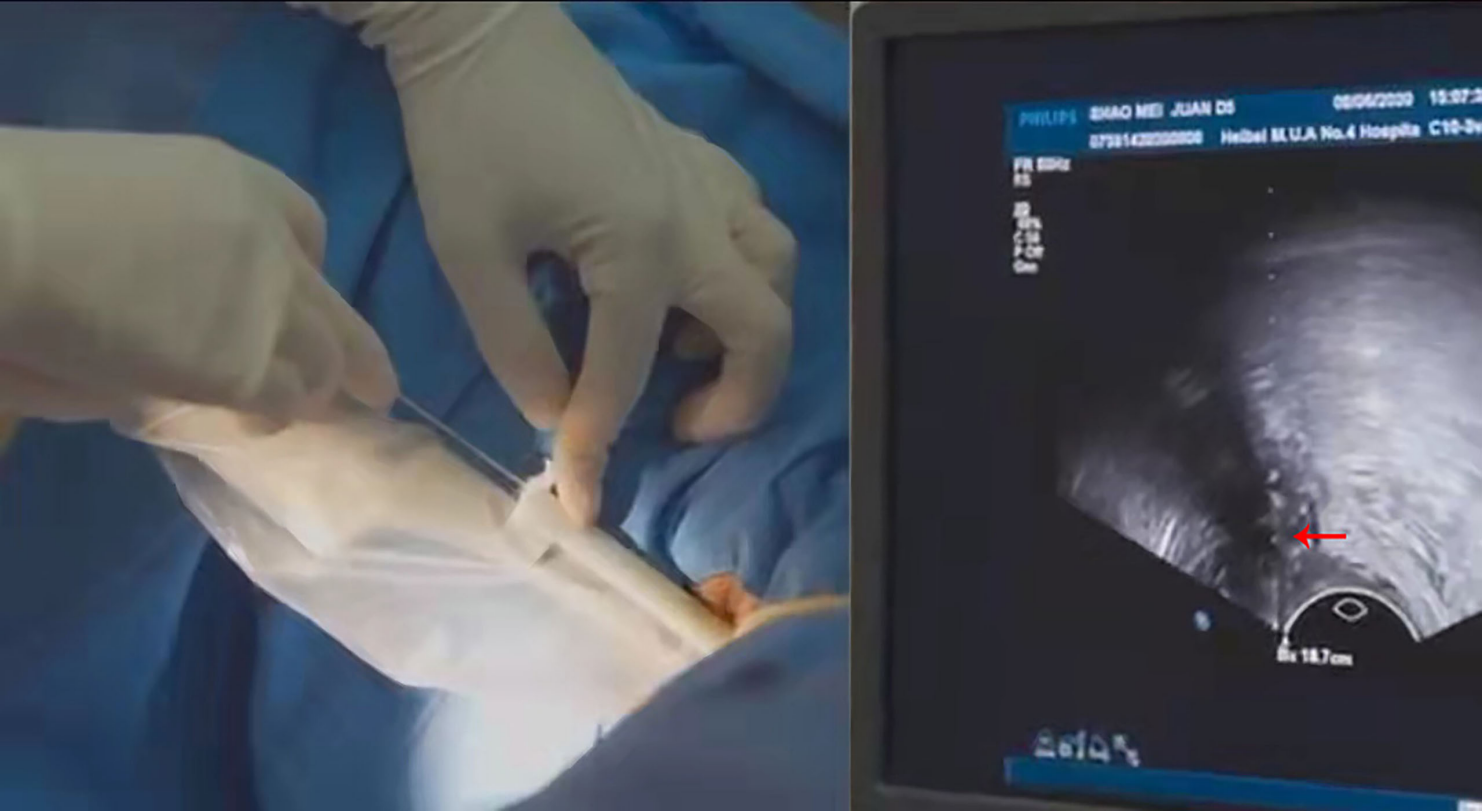
Implant therapy with the puncture bracket under transvaginal ultrasound guidance.

The puncture bracket is suitable for use with an implant needle with a diameter of 1.5 mm and a length of 20 cm. In clinical treatment, the US doctor first adjusts the US instrument to the puncture guide state, places a US probe with a puncture bracket (**Figure 2**) in the patient’s vagina, and adjusts the direction of the probe to find the needle entry path through which the pelvic masses can be clearly displayed and blood vessels and important organs can be avoided. Then, the surgeon places the needle into the needle passage of the puncture bracket and directly places the needle into the tumor tissues to the top boundary of the tumor along the US puncture guide line. After the implant is completed, the surgeon lifts the cover plate upward, and the sonographer presses the ultrasonic probe gently in the opposite direction at the same time, so that the needle can be easily separated from the puncture bracket. The surgeon can withdraw the probe outwards, reinstall the cover plate, reposition the needle, and place it back into the vagina several times. The tumor size was evaluated by MRI, CT and ultrasound before surgery. Approximate tumor volume and the number of implanted needles required before implantation therapy. For larger tumors, this method can be used to place multiple needles into the tumor tissues through the vagina.

## Clinical application

### Patient characteristics

Between 2020 and 2021, a total of 56 patients with gynecological oncology received real-time transvaginal US-guided implant therapy (**Table 1**). The indications for ISBT were as follows: 1) patients with pelvic relapse after gynecological oncology surgery who still had residual pelvic mass after completing pelvic external beam radiation therapy (EBRT); 2) patients with cervical cancer who had completed standard EBRT treatment without sufficient regression of the parametrial invaded tissues; and 3) patients with recurrence in the radiation field after a previous standard treatment course of pelvic radiotherapy. The treatment schedules were as follows: 1) patients received EBRT + ISBT treatment, consisting of whole pelvis EBRT with a dose of 45–50 Gy with conventional fractionation (1.8–2.0 Gy), followed by two ISBT implant sessions one week with an accumulated dose of 18–28 Gy. The cumulative radiotherapy dose (equivalent dose in 2 Gy [EQD2]) was 80–100 Gy after conversion; 2) patients who had previously received pelvic cavity radiotherapy only received ISBT treatment in the second radiotherapy, comprising one weekly session of ISBT implant with a dose of 7 Gy and an accumulated dose of 40–60Gy after EQD2 conversion. Weekly chemotherapy with cisplatin 40 mg/m2 was also given to suitable patients who with ECOG (Eastern Cooperative Oncology Group) score of 1-2 during EBRT.

**Table 1:** Patient characteristics.

Characteristics	Median (range)	n = 56 (100%)	
		n	%
treatment age	61 (35-71)		
FIGO stage (cervical cancer)			
IA		2	3.6
IB		5	8.9
IIA		6	10.7
IIB		16	28.6
IIIB		18	32.1
IVA		3	5.4
FIGO stage (endometrial cancer)			
IA		1	1.8
IB		1	1.8
II		0	0
IIIA		0	0
IIIB		0	0
IIIC1		3	5.4
IIIC2		1	1.8
Maximum recurrence tumor diameter (cm) before ISBT			
<4 cm		12	21.4
≥4 cm		7	12.5
Histology			
Squamous carcinoma		47	83.9
Adenomatous carcinoma		9	16.1
Pathology details			
Grade1-2		21	37.5
Grade3		30	53.6
Unclassfied		5	8.9
Hemoglobin before ISBT (g/L)			
<7		4	7.1
≥7		52	92.9
Time to postoperative recurrence(mouth)			
<6		5	8.9
≥6		14	25
Postoperative recurrence site			
Central recurrence		8	14.3
Peripheral recurrence		11	19.6
Previous RT			
Yes		5	8.9
no		51	91.1
EBRT modility (Gy)			
ISBT only		5	8.9
ISBT+EBRT		51	91.1
EQD2 (Gy) Median			
<85		11	19.6
≥85		45	80.4
Chemo			
With chemo		52	92.9
Without chemo		4	7.1

ISBT, interstitial brachytherapy; EBRT, external beam radiotherapy; EQD2, equivalent dose in 2 Gy fractions; FIGO, The International Federation of Gynecology and Obstetrics; SCC, squamous-cell carcinoma antigen.

### The ISBT treatment procedure

Before the implant therapy, the patients underwent a transvaginal US assessment. Enteroclysis was performed the night before and on the day of treatment to reduce the interference of feces with the US. The patient was put in the lithotomy position during treatment. The vulva and vagina were routinely disinfected, and an indwelling catheter was used to inject 60–100ml normal saline into the bladder, facilitating transabdominal US observation of the pelvic structure. A sterile isolation sleeve was placed over the ultrasonic intracavity probe, and then the puncture bracket was placed on top of the sterile sleeve and fixed onto the probe. The US probe was placed into the vagina, clearly displaying the size, shape, relationship of the tumor to surrounding tissues, and the vascular route, which could then be observed and evaluated. In order to obtain better target area conformability, after designing the position, number and safe approach path of the implant needles, the operator placed the implant needles into the tumor tissue along the ultrasound-guided line through the needle passage in the puncture bracket to the target tumor area. The optimal position of the needle tip was observed in real time using the US (**Figure 2**) and the desired number of needles were inserted for adequate coverage of the target area. After US-guided puncture and needle placement, image-guided brachytherapy was planned using Oncentra Brachy. The CT scan with a layer of 3mm is used for precise positioning, and the CT image obtained by the scan is transmitted to the treatment planning workstation through the network. Doctors determine target volume and risk organs based on CT images. At the same time, combined with whether there is abnormal bleeding after needle extraction, check whether there is damage to adjacent organs during the operation.

## Results

All the patients were generally speaking in good health at the time of relapse although six of them had complained of sciatica. The period of time from the beginning of the external radiation therapy to the end of the BT was no more than eight weeks, the range being from four to eight weeks. All the treatment procedures went to plan, and the average duration of the implant operation was 15 min (7–31 min). The cumulative radiotherapy dose of external irradiation combined with BT was 80–100 Gy. Thirteen of the patients in this study, who received implant therapy, had recurrent pelvic tumors after cervical cancer surgery and six had recurrent pelvic tumors after endometrial cancer surgery, and five of the 19 patients had recurrent tumors in the radiation field where they had received prior radiotherapy. The other 37 cases were locally advanced cervical cancer patients. Patients who relapsed in the pelvic radiation field only received ISBT treatment, but all the others received standard EBRT therapy initially and then ISBT, as well as concurrent chemotherapy with cisplatin.

EQD2 at the ISBT implantation site was 88 Gy (range 80–00 Gy). A total of 736 needles were placed in the 56 patients under transvaginal US guidance, and the satisfaction rate of implantation was as high as 91%. Some needles were considered to be ineffective because the spacing between the needles was too close for all the needle passages to provide an effective dose. Postoperative pelvic recurrent lesions in 8 cases were located in the center of the pelvic cavity, and the lesions in 11 cases were located in the lateral pelvic cavity. The median lesion volume was 37.7 cm^3^ (range 2.6–237.8 cm^3^). No side effects, such as perforation of the bladder or bowel or severe bleeding, occurred during the operation (see **Table 2**).

**Table 2:** Recommended and achieved dose–volume parameters for ISBT.

Dose–volume parameters	Recommended	Achieved		
		3 fraction	4 fraction	5fraction
CTV (cm^3^)		8.15	42.53	113.53
Catheters		4	8	10
V100 (%)	≥90	96.5 (92-99.1)	91.4 (62.8-99.3)	89.8 (46.3-98.1)
D90 (%)	≥100	104.1 (90.1-117.9)	101.3 (85.5-113.2)	98.1 (70.8-111.6)
Rectum D2cc (Gy)	≤75	71.2 (53.5-80.1)	73.1 (62.3-79.8)	74.9 (59.3-81.2)
Bladder D2cc (Gy)	≤95	83.2 (61.1-93.2)	85.4 (72.3-94.2)	91.3 (81.8-96.9)
The small intestine D2cc (Gy)	≤75	61.2 (42.5-73.1)	71.1 (59.3-78.8)	73.2 (62.3-81.2)
COIN		0.72 ± 0.02	0.61 ± 0.01	0.53 ± 0.01

All achieved parameters are presented as median (range).

V100, volume receiving 100% of the prescribed dose; D90, dose received by 90% volume of HRCTV; COIN, conformity index.

**Figure 3:**
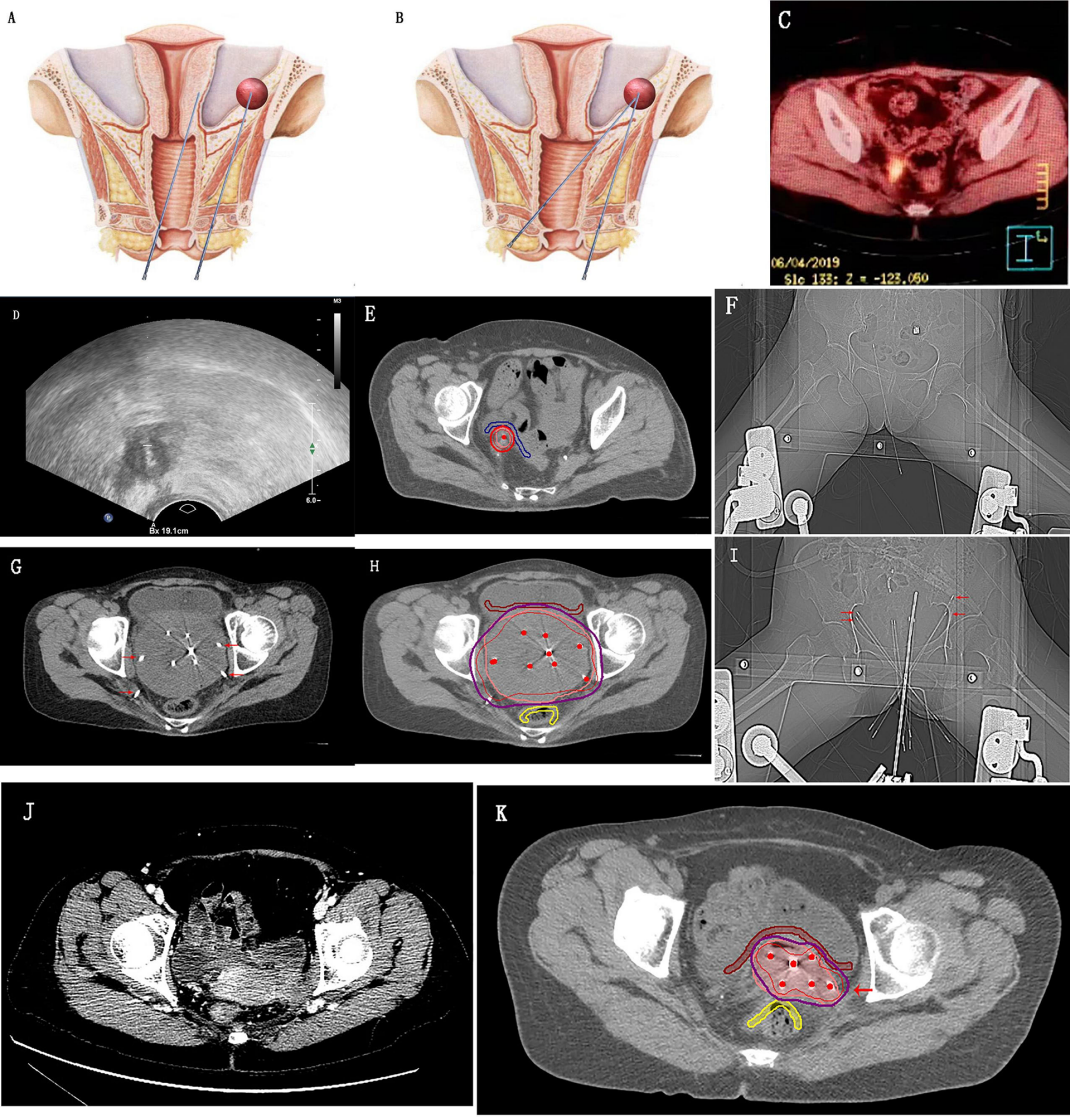
Transvaginal needle placement with the detachable puncture bracket. **(A, B)** Schematic diagrams of the pelvic cavity comparing the length of paths of transvaginal and perineal needle insertion; **(C–F)** Positron emission tomography/computed tomography (CT) shows recurrent lesions adjacent to the right ureter in the pelvic radiation field after cervical cancer surgery; ultrasound shows real-time ultrasound-guided transvaginal insertion of a needle into the tumor tissues; CT shows an accurate implant result and highly conformal high-risk clinical target volume; **(G–I)** CT shows the large pelvic mass implant; **(J, K)** Implant therapy for the periuterine invasion of cervical cancer. The needles indicated by the red arrow in the figure are the sites that can be implanted using a detachable puncture bracket under transvaginal ultrasound real-time guidance.

**Figure 3** shows implants in the lateral wall area of the pelvic cavity, beside the ureter, large pelvic masses, and the parametrial invasion area of cervical cancer. CT images were used to verify the accuracy and safe use of the needle.

## Discussion

One study by the International Federation of Gynecology and Obstetrics showed that the relapse rates of cervical cancer patients in Phases Ib and IIa were 10% and 17% respectively, and 35% of the postoperative relapse lesions were located in the pelvic cavity. The relapse rates of patients in Phases IIb, III, and IVa were 23%, 42%, and 74%, respectively (10–12). While some patients with locally relapsed or localized metastatic disease can be cured through surgery or radiotherapy, most patients cannot. The 5-year overall survival (OS) rate of patients with cervical cancer relapse is only 3.2%–16.5% (13–15), and that of patients with endometrial cancer relapse is 40% (16). Improving the therapeutic effect in patients with gynecological oncology relapse and patients with local advanced cervical cancer through the improvement of radiotherapy technology is an issue that radiotherapists are eager to address. BT plays a very important role in the treatment of gynecological tumors (17). For patients with large tumors (diameter >4 cm), or severe parametrial invasion, as well as recurrent tumors located in various parts of the pelvic cavity, the intracavitary BT technique cannot be used to give high-dose radiation to the target area, and it is better to use implants. With the widespread development of image-guided interstitial implant radiotherapy in recent years, more and more patients who need elevation of the radiotherapy dose for a local tumor, which cannot be achieved in traditional two-dimensional or three-dimensional BT, have had the opportunity of being cured (18, 19). However, the coverage of the ISBT target area of some tumors is not always adequate due to the range of difficulty in implant therapy in different parts of the tumor and in the doctors’ skills. In order to protect the organs involved, the need for precision is even greater when radiotherapy is chosen a second time for the treatment of recurrent tumors in the radiation field. Thus, improving the precision of implant radiotherapy is the key to addressing the issue of the conformal degree of tumor covered by the radiotherapy target area, and it is a clinical problem that needs an urgent solution. Even if the image-guided 3D interstitial implant radiotherapy is used, it is still difficult to achieve visual implantation under accurate and real-time transvaginal guidance. Bare-handed transperineal or transvaginal implants require the surgeon to choose the needle entry position and angle based on their experience and with reference to the physical examination and imaging evaluation of the patient. This requires a highly-skilled surgeon, and it can be difficult for even experienced clinicians to perform the operation with precision every time, and repeatability tends to be poor. In addition, there is a lag in verifying whether the needle is in place, and the needles may have to be removed and replaced or further adjusted as required. For tumors located in the center of the pelvic cavity, there is also a higher risk of damage to the bladder and rectum because of their proximity. 3D-PNCT positioning and implant technology involves relatively complicated procedures, the operation is time-consuming, usually requiring anesthesia, and the needle path cannot be freely chosen. Since blind bare-handed implant technology has limited accuracy and poor repeatability, it is difficult to widely promote this technology in clinical practice.

It has been found that that the main reason for the inability of transvaginal insertion of the needle around the pelvic cavity during clinical treatment is that the possibility of vaginal extension and torsion is limited by the use of a vaginal speculum. Thus, even the maximum tilt angle cannot allow for needle entry from the vagina into the lateral wall area of the pelvic cavity. However, without the speculum, vagina extension and torsion are possible, and it was these characteristics of the vagina that led to the development of a puncture bracket that can be guided by real-time vaginal US and separated from the needle. Transvaginal US was used as a guide because intravaginal US is closer to the tumor in the pelvic cavity than abdominal US, with a shorter puncture path and a smaller risk of damage. Moreover, the probe has a higher resolution ratio and sharper images. The US probe can display all the pelvic structures in the area of interest in real time during implantation, and the surgeon can place the needle into the target area under real-time visualization (9). The sagittal section of the US probe is crucial in the implantation process because it can show the optimal puncture path, and the images can also be used to assess the overall length of the target area, which will help determine the length of the needle. Transabdominal US cross-section imaging is performed alternately during the process to ensure that the needle is covering the target area and that it does not enter the bladder, rectum, or small intestine. The intraluminal ultrasound probe can visualize the border of the pelvic mass at a depth of 7 to 8 cm from the top of the probe, as well as the bowel and blood vessels near the mass. During the operation, the needle insertion route of blood vessels and organs at risk will be avoided, and the implantation needle will be placed near the tumor boundary without penetrating the tumor tissue. In addition, the separation of the puncture bracket and the needle can be done after the needle is inserted into the tumor tissues so that the subsequent BT can be completed. This technology can avoid the complex pre-planning of 3D-PNCT technology or the uncertainty of bare-handed implantation. More importantly, a precise transvaginal implant for masses in the lateral wall area in the pelvic cavity can be achieved with the shortest path. It has been reported that transrectal US real-time guidance was used in the implant treatment of gynecological oncology, while relying on Syed template implant technology. This method requires a speculum in the operation process, but it is also faced with the problem that some masses around the pelvic cavity cannot be treated with transvaginal implantation. Besides this, the operation is relatively complex and so the Syed template and needle insertion may take an additional 30 to 120 min, which means the whole operation reportedly takes 45 to 165 min (20). However, the new detachable puncture bracket for the transvaginal US-guided implant technology significantly shortens the operation time and makes the operation more feasible.

It has been observed that the advantages of transvaginal needle insertion over transperineal needle insertion are as follows: 1) the path of the needle is much shorter, the patient feels little pain, and there is no need for anesthesia (**Figure 3**); 2) with a high degree of precision, masses larger than 5 mm can be shown using US, and the positioning puncture can be completed in a visualized manner under real-time guidance; 3) it is easy to perform so general resident doctors can carry out the therapy with the assistance of US doctors; and 4) the safety of the implant therapy is greater, and the likelihood of injury to adjacent organs is considerably lower. CT-guided 3D-PNCT therapy does not compare favorably with transvaginal needle insertion for the following reasons: 1) 3D-PNCT technology requires a treatment plan, which is time-consuming; 2) the cost of 3D-PNCT is high, and the operation is relatively complex; 3) anesthesia is required, and the needle inserting route cannot be freely chosen; 4) real-time operation cannot be achieved; and 5) the positioning advantage for smaller lesions is inferior.

In this study, 56 patients meeting the conditions for implant therapy were chosen, including patients with a local cervical tumor diameter greater than 4 cm after external irradiation, severe parametrial invasion, and pelvic relapse after gynecological oncology. A total of 736 needles were inserted under transvaginal US real-time guidance with an intracavity probe puncture bracket. The average duration of implantation was 15 min (7–31 min), and the cumulative dose of external irradiation combined with BT was 80–100 Gy. The arrangement effect of all the needles was assessed, and the satisfaction rate of implantation was as high as 91%. Certain needles were considered to be ineffective because the spacing between the needles was too close for all the needle passages to provide an effective dose. There were no side effects, such as perforation of the bladder or bowel or severe bleeding, during the operation. For small tumors, ISBT is usually administered once a week, totaling 3–4 sessions of ISBT treatment. Due to the small size of the tumor and the high precision of the implant, the mean values of V (volume) 100 and D (dose) 90 for each treatment at the dose tolerable to the adjacent organ met the requirements of the prescription plan. For patients who received five sessions of ISBT treatment, the V100 of some patients failed to meet the requirement of ≥90%, usually due to the large size of the tumor or its location close to the rectum and bladder. While magnetic resonance imaging (MRI) and US can show the structure of a tumor in 3D and clearly display the extent of a cervical lesion, it is difficult for CT to accurately distinguish the size and shape of a tumor, and it cannot accurately delineate the upper boundary of any cervical cancer, which means there may be overestimation with respect to the left and right sides of the tumor (21). MRI is used routinely in the diagnosis of cervical cancer due to its increased sensitivity compared to CT, and its use in gynecologic brachytherapy planning is also gradually increasing (22). At present, MRI images are used in most studies of intracavitary radiotherapy around the world, and the European Society for Radiotherapy and Oncology and the American Society for Radiation Oncology recommendations are also based on MRI. Nevertheless, with respect to the current radiotherapy procedures used in China, for reasons of economy and convenience, CT is still the most commonly used method. Thus, the high-risk clinical target volume delineated in this study may have been larger than the actual tumor volume, and the actual ISBT parameters, including the conformal degree of the target area and the actual tumor volume irradiated at the prescribed dose, may have been better than the results shown in the data analysis.

To sum up, the use of interstitial implant technology under transvaginal US guidance eliminates both the inaccuracy of inserting needles without visual guidance and the risk of injury to adjacent organs. It also overcomes the time-consuming problem of pre-planning experienced with the use of CT or MRI. At the same time, the best and shortest path can be selected to achieve high-precision implant therapy. This treatment method also allows for the placement of multiple needles, is simple to operate, and eliminates the need for anesthesia. It is now necessary to investigate how a more effective placement of needles in 3D can be made possible so that larger and more irregularly-shaped masses can be treated successfully using this technique.

## Data availability statement

The original contributions presented in the study are included in the article/supplementary material. Further inquiries can be directed to the corresponding author.

## Ethics statement

The studies involving human participants were reviewed and approved by Fourth Hospital of Hebei Medical University ethics committee. The patients/participants provided their written informed consent to participate in this study.

## Author contributions

Conception and design of the research, HL. Acquisition of data, S-GW, S-HN, H-XN. Analysis and interpretation of the data, X-JY. Statistical analysis, XC. Obtaining financing, HL. Writing of the manuscript, HL and X-JY. Critical revision of the manuscript for intellectual content, YY. All authors contributed to the article and approved the submitted version.

## Funding

Natural Science Foundation of Hebei Province (H2021206429).

## Conflict of interest

The authors declare that the research was conducted in the absence of any commercial or financial relationships that could be construed as a potential conflict of interest.

## Publisher’s note

All claims expressed in this article are solely those of the authors and do not necessarily represent those of their affiliated organizations, or those of the publisher, the editors and the reviewers. Any product that may be evaluated in this article, or claim that may be made by its manufacturer, is not guaranteed or endorsed by the publisher.

## References

[1] da Silva VTM Fortuna Diniz AP Martins J Cursino K Esteves SCB Teixeira JC. Use of interstitial brachytherapy in pelvic recurrence of cervical carcinoma: Clinical response, survival, and toxicity. Brachytherapy (2019) 18(2):146–53. doi: 10.1016/j.brachy.2018.11.002 30591409

[2] Yoshida K Yamazaki H Kotsuma T Takenaka T Masui K Yoshioka Y. Treatment results of image-guided high-dose-rate interstitial brachytherapy for pelvic recurrence of uterine cancer. Brachytherapy (2015) 14(4):440–8. doi: 10.1016/j.brachy.2015.02.195 25858904

[3] Murakami N Kobayashi K Shima S Tsuchida K Kashihara T Tselis N. A hybrid technique of intracavitary and interstitial brachytherapy for locally advanced cervical cancer: initial outcomes of a single-institute experience. BMC Cancer (2019) 19(1):221. doi: 10.1186/s12885-019-5430-x 30866877 PMC6417107

[4] Mohamed S Kallehauge J Fokdal L Lindegaard JC Tanderup K. Parametrial boosting in locally advanced cervical cancer: combined intracavitary/interstitial brachytherapy vs. intracavitary brachytherapy plus external beam radiotherapy. Brachytherapy (2015) 14(1):23–8. doi: 10.1016/j.brachy.2014.09.010 25455382

[5] Sekii S Tsujino K Kosaka K Yamaguchi S Kubota H Matsumoto YN. Inversely designed, 3D-printed personalized template-guided interstitial brachytherapy for vaginal tumors. J Contemp Brachyther (2018) 10(5):470–7. doi: 10.5114/jcb.2018.78832 PMC6251441 30479625

[6] Wang Y Ye WJ Du LH Li AJ Ren YF Cao XP. Dose-volume parameters and clinical outcome of CT-guided free-hand high-dose-rate interstitial brachytherapy for cervical cancer. Chin J Cancer (2012) 31(12):598–604. doi: 10.5732/cjc.011.10452 22640625 PMC3777456

[7] Campelo S Subashi E Meltsner SG Chang Z Chino J Craciunescu O. Multimaterial three-dimensional printing in brachytherapy: Prototyping teaching tools for interstitial and intracavitary procedures in cervical cancers. Brachytherapy (2020) 19(6):767–76. doi: 10.1016/j.brachy.2020.07.013 PMC8488976 32893145

[8] Aggarwal V Chuprin A Aggarwal A Vingan H Crandley E. Bleeding after interstitial brachytherapy for cervical cancer requiring embolization. Radiol Case Rep (2018) 13(6):1141–5. doi: 10.1016/j.radcr.2018.07.033 PMC6138866 30233746

[9] Siebert FA Kirisits C Hellebust TP Baltas D Verhaegen F Camps S. GEC-ESTRO/ACROP recommendations for quality assurance of ultrasound imaging in brachytherapy. Radiother Oncol (2020) 148:51–6. doi: 10.1016/j.radonc.2020.02.024 32335363

[10] Kasamatsu T Onda T Yamada T Tsunematsu R. Clinical aspects and prognosis of pelvic recurrence of cervical carcinoma. Int J Gynaecol Obstet (2005) 89(1):39–44. doi: 10.1016/j.ijgo.2004.12.020 15777897

[11] Peiretti M Zapardiel I Zanagnolo V Landoni F Morrow CP Maggioni A. Management of recurrent cervical cancer: a review of the literature. Surg Oncol (2012) 21(2):e59–66. doi: 10.1016/j.suronc.2011.12.008 22244884

[12] Pfaendler KS Tewari KS. Changing paradigms in the systemic treatment of advanced cervical cancer. Am J Obstet Gynecol (2016) 214(1):22–30. doi: 10.1016/j.ajog.2015.07.022 26212178 PMC5613936

[13] Legge F Chiantera V Macchia G Fagotti A Fanfani F Ercoli A. Clinical outcome of recurrent locally advanced cervical cancer (LACC) submitted to primary multimodality therapies. Gynecol Oncol (2015) 138(1):83–8. doi: 10.1016/j.ygyno.2015.04.035 25940427

[14] Lai CH. Management of recurrent cervical cancer. Chang Gung Med J (2004) 27(10):711–7. 15646293

[15] Ferlay J Steliarova-Foucher E Lortet-Tieulent J Rosso S Coebergh JWW Comber H. Cancer incidence and mortality patterns in Europe: estimates for 40 countries in 2012. Eur J Cancer (2013) 49(6):1374–403. doi: 10.1016/j.ejca.2012.12.027 23485231

[16] Khoury-Collado F Einstein MH Bochner BH Kaled M Alektiar KM Sonoda Y. Pelvic exenteration with curative intent for recurrent uterine malignancies. Gynecol Oncol (2012) 124(1):42–7. doi: 10.1016/j.ygyno.2011.09.031 22014627

[17] Kanaev SV Turkevich VG Baranov SB Savel’eva VV. [Basic principles and results of brachytherapy in gynecological oncology]. Vopr Onkol (2014) 60(4):422–8. 25552060

[18] Nesvacil N Schmid MP Pötter R Kronreif G Kirisits C. Combining transrectal ultrasound and CT for image-guided adaptive brachytherapy of cervical cancer: Proof of concept. Brachytherapy (2016) 15(6):839–44. doi: 10.1016/j.brachy.2016.08.009 27693172

[19] Schmid MP Nesvacil N Pötter R Kronreif G Kirisits C. Transrectal ultrasound for image-guided adaptive brachytherapy in cervix cancer - an alternative to MRI for target definition? Radiother Oncol (2016) 120(3):467–72. doi: 10.1016/j.radonc.2016.01.021 26921168

[20] Stock RG Chan K Terk M Dewyngaert JK Stone NN Dottino P. A new technique for performing syed-neblett template interstitial implants for gynecologic malignancies using transrectal-ultrasound guidance. Int J Radiat Oncol Biol Phys (1997) 37(4):819–25. doi: 10.1016/s0360-3016(96)00558-5 9128957

[21] Viswanathan AN Dimopoulos J Kirisits C Berger D Pötter R. Computed tomography versus magnetic resonance imaging-based contouring in cervical cancer brachytherapy: results of a prospective trial and preliminary guidelines for standardized contours. Int J Radiat Oncol Biol Phys (2007) 68(2):491–8. doi: 10.1016/j.ijrobp.2006.12.021 17331668

[22] Kapur T Egger J Damato A Schmidt EJ Viswanathan AN. 3-T MR-guided brachytherapy for gynecologic malignancies. Magn Reson Imaging (2012) 30(9):1279–90. doi: 10.1016/j.mri.2012.06.003 PMC3468320 22898699

